# Editorial: Quantitative neuroradiology methods

**DOI:** 10.3389/fradi.2024.1366704

**Published:** 2024-02-12

**Authors:** Mojtaba Barzegar, Mark Schweitzer, Tiffany Y. So, Yongsheng Chen, Şükrü Mehmet Ertürk

**Affiliations:** ^1^ Radiation-Oncology Department, National Center for Cancer Care and Research (NCCCR), Hamad Medical Corporation (HMC), Doha, Qatar; ^2^ Society for Brain Mapping and Therapeutics, Los Angeles, CA, United States; ^3^ Intelligent Quantitative Bio-Medical Imaging (IQBMI), Tehran, Iran; ^4^ Office of the Vice President for Health Affairs Office of the Vice President, Wayne State University, Detroit, MI, United States; ^5^ Department of Imaging and Interventional Radiology, The Chinese University of Hong Kong, Hong Kong, Hong Kong SAR, China; ^6^ Department of Neurology, Wayne State University School of Medicine, Detroit, MI, United States; ^7^ Department of Radiology, Faculty of Medicine, İstanbul University, Istanbul, Turkiye

**Keywords:** motion artefact, DKI-diffusion kurtosis imaging, BCVI-blunt cerebrovascular injury, CNN—convolutional neural network, ethics—clinical

**Editorial on the Research Topic** Quantitative neuroradiology methods

Neuroradiology has been and continues to be one of the fastest advancing imaging subspecialties, often driven by breakthroughs in artificial intelligence (AI) and pulse sequence advancements. These converging technologies hold immense promise for minimizing artifacts, leading to markedly improved diagnostic accuracy, as well as improved treatment decisions and prognostication.

Despite the increasing speed of MRI, motion remains a problem, particularly in younger subjects. Children's head motion during magnetic resonance imaging (MRI) poses a significant challenge, impacting image quality and diagnostic reliability. Analyzing motion patterns in pediatric patients with and without general anesthesia reveals differences, emphasizing the necessity of tailored approaches to mitigate artifacts and optimize image acquisition, and underscoring the importance of refining imaging protocols for enhanced clinical outcomes. The article by Eichhorn et al. presents relevant insights on motion artefacts and clinical implications.

In the very youngest patients, these motion mitigation techniques allow new avenues to be explored. Among these is the imaging of neonatal patients. Despite challenges in its application, diffusion kurtosis imaging (DKI) offers a unique window into the intricate microstructural changes within the neonatal spinal cord. Proposing a novel semi-automated pipeline for neonatal spinal cord imaging, Trò et al. illuminate DKI's superior sensitivity over traditional diffusion imaging methods, emphasizing its potential in elucidating early developmental anomalies and brain-spinal cord interconnections critical for long-term outcomes, and minimization of societal burden.

As patients mature, different types of disorders become prevalent. In the realm of blunt cerebrovascular injuries (BCVIs), emergent imaging modalities have transformed the landscape of detection and characterization. Evolving techniques, such as advanced magnetic resonance imaging (MRI) sequences and transcranial Doppler, have markedly improved the diagnosis and understanding of BCVIs, heralding a new era of precision imaging for trauma-related neurovascular injuries for these increasingly prevalent injuries (Bounajem et al.).

One of the first applications of AI, has been in image segmentation. AI has traditionally been limited by the size, and diversity of the databases, the networks are adapted from. Advances in weakly-supervised segmentation methodologies leveraging convolutional neural networks (CNNs) presents a potential ethical solution to the challenge of limited annotated datasets in medical imaging. The proposed pipeline by Rajapaksa and Khalvati utilizes relevance maps and localized perturbations, demonstrating enhanced accuracy in glioma brain tumor segmentation and showcases the potential of ethical AI solutions in neuroimaging tasks.

There are advances in MRI sequences, image processing, and data interpretation. The latter is where AI is most beneficial. AI, understandably is much in the news nowadays. The integration of AI in neuroradiology promises unparalleled insights into brain pathophysiology, refining treatment models, and augmenting diagnostic algorithms. Yet, this autonomous utilization raises critical ethical challenges. Issues of informed consent scope, data privacy risks, database biases, and the ethical responsibility accompanying AI-driven decisions cast a spotlight on the ethical landscape of neuroscience research and healthcare practices. The ethics of AI deserve far more attention in the medical literature, and we hope this issue will help guide that conversation (Khosravi and Schweitzer).

We hope with this editorial and Research Topic to synthesize the ethical dilemmas and technical innovations in neuroimaging advances and emphasize the necessity of establishing cohesive ethical standards. A balanced approach is advocated, one that harnesses the potential of advanced technologies while safeguarding patient privacy, ensuring informed consent, and considering database biases. By doing so, we prioritize ethical responsibilities in the pursuit to enhance the field of neuroimaging.

